# The Prognostic Role of Geriatric Nutritional Risk Index in Periampullary Cancer Patients Undergoing Pancreaticoduodenectomy: A Propensity Score-Matched Survival Study

**DOI:** 10.3390/cancers17193273

**Published:** 2025-10-09

**Authors:** Chih-Ying Li, Wei-Feng Li, Yueh-Wei Liu, Yu-Yin Liu, Cheng-Hsi Yeh, Yu-Hung Lin, Jen-Yu Cheng, Shih-Min Yin

**Affiliations:** 1 Division of General Surgery, Department of Surgery, Kaohsiung Chang Gung Memorial Hospital, Chang Gung University College of Medicine, Kaohsiung 83301, Taiwan; u105001023@cgmh.org.tw (C.-Y.L.); webphone@cgmh.org.tw (W.-F.L.); anthony0612@cgmh.org.tw (Y.-W.L.); liuyuyin@cgmh.org.tw (Y.-Y.L.); ycc9002108@cgmh.org.tw (C.-H.Y.); adrianlin107@cgmh.org.tw (Y.-H.L.); 2 Weight Management Center, Kaohsiung Chang Gung Memorial Hospital, Chang Gung University College of Medicine, Kaohsiung 83301, Taiwan; 3 Department of Radiation Oncology & Proton and Radiation Therapy Center, Kaohsiung Chang Gung Memorial Hospital, Chang Gung University College of Medicine, Kaohsiung 83301, Taiwan; york480@cgmh.org.tw

**Keywords:** nutrition assessment, pancreaticoduodenectomy, periampullary neoplasms, treatment outcome, survival

## Abstract

**Simple Summary:**

Malnutrition is common in patients with periampullary cancers and can negatively affect recovery and survival after major surgery. This study focused on the Geriatric Nutritional Risk Index (GNRI), a simple tool that uses basic health data to assess a patient’s nutrition status before surgery. This study reviewed medical records of patients who underwent pancreaticoduodenectomy using the largest private healthcare database in Taiwan, which found that patients with poor GNRI scores had shorter survival, more post-operative complications, and higher short-term death rates. These results were consistent in both pancreatic and other nearby cancers. Our findings suggest that GNRI may be a practical and cost-effective way to identify high-risk patients before surgery.

**Abstract:**

**Background:** The Geriatric Nutritional Risk Index (GNRI) is a simple tool for nutritional assessment, but its long-term prognostic value in patients undergoing pancreaticoduodenectomy (PD) remains unclear. **Methods**: This retrospective study included adult patients who underwent PD between January 2014 and December 2023 at Chang Gung Memorial Hospital. Patients were grouped by GNRI: inferior (<82), moderate (82–98), and superior (≥98). Propensity score matching was performed based on age, sex, cancer type, surgical approach, and ASA status. Primary outcomes were overall survival (OS) and recurrence-free survival (RFS). **Results**: Among 371 patients, inferior GNRI was associated with worse median survival time (18.64 vs. 34.62 months, HR = 2.953, *p* < 0.001). This association was observed in both pancreatic cancer and other periampullary malignancies. Inferior GNRI also correlated with higher short-term mortality and adverse perioperative outcomes, including longer ICU stay, and greater need for ventilator support, reintubation, reoperation and total parenteral nutrition (TPN). **Conclusions**: Preoperative GNRI is a strong predictor of survival and short-term outcomes in PD patients. Early nutritional assessment may aid risk stratification and intervention.

## 1. Introduction

Periampullary neoplasms remain a devastating disease despite advancements in medical treatments. Patients with these malignancies often experience significant nutritional deficits due to tumor-related metabolic disturbances, exocrine insufficiency, and impaired gastrointestinal function [1]. In many cases, particularly among older adults, these tumors contribute to reduced oral intake and nutrient malabsorption caused by biliary or gastrointestinal obstruction [2]. Studies have shown that such nutritional imbalances are associated with poor clinical outcomes, including reduced overall survival and an increased risk of adverse events [2,3,4,5,6].

Pancreaticoduodenectomy (PD) is one of the most complex surgical procedures for managing periampullary lesions. The surgery involves the removal of the duodenum, pancreatic head, bile duct, and sometimes the gastric pylorus, all of which are essential for nutrient absorption [2]. Despite advancements in surgical techniques that have reduced mortality rates, PD continues to carry a high risk of morbidity [7]. Post-operative complications such as surgical site infections, delayed gastric emptying, and pancreatic fistulas are common and occur more frequently in patients with preoperative malnutrition [3,8]. Research indicates that poor nutritional status before surgery increases the risk of surgical complications and mortality, highlighting the need for thorough preoperative nutritional assessment and timely intervention [4,9].

Several screening tools have been developed to identify patients at risk of malnutrition, including the Subjective Global Assessment (SGA), Malnutrition Universal Screening Tool (MUST), Mini Nutritional Assessment (MNA), Nutritional Risk Screening Tool 2002 (NRS-2002), Geriatric Nutrition Risk Index (GNRI), Prognostic Nutritional Index (PNI), and Controlling Nutritional Status (CONUT) score [10,11]. Although some tools are more accurate [12], they often require extensive history-taking and rely on subjective parameters, limiting their practicality in busy clinical settings [13]. GNRI is a simple, cost-effective nutritional assessment tool that incorporates serum albumin levels and the ratio of actual to ideal body weight. Owing to its simplicity, GNRI is a promising option for preoperative nutritional evaluation; however, its application in patients undergoing PD remains unexplored.

This study aimed to evaluate the prognostic value of the GNRI as a practical tool for predicting short- and long-term post-operative outcomes in patients undergoing PD for periampullary neoplasms using the Chang Gung Research Database (CGRD), the largest private healthcare database in Taiwan.

## 2. Materials and Methods

### 2.1. Patient

This study retrospectively reviewed data from patients in the Chang Gung Research Database (CGRD) who underwent pancreaticoduodenectomy (PD) between January 2014 and December 2023. The CGRD, managed by Taiwan’s largest healthcare provider, contains data from seven medical institutions across the country. All medical records in the database were de-identified to ensure that no individual could be identified directly or indirectly. Patients without GNRI data, with missing surgical records or younger than 18 years of age were excluded. This study was approved by the Institutional Review Board of Chang Gung Memorial Hospital (IRB No.: 202500746B0), and all procedures were conducted in accordance with its guidelines and regulations. The Institutional Review Board waived the requirement for informed consent.

Clinicopathological data were collected from medical records. The following parameters were analyzed: (1) demographic data and hospital stay including age, sex, height, weight, admission date, hospital stay duration, and intensive care unit (ICU) stay duration; (2) comorbidities such as hypertension, diabetes mellitus and ASA physical status classification; (3) laboratory parameters, including CA19-9, albumin and total bilirubin levels; (4) surgical details, including operative duration, estimated intraoperative blood loss, and surgical approach (open or minimally invasive); (5) pathological findings, including histological type, number of lymph nodes extracted, number of lymph nodes involved, tumor size and margin status; (6) preoperative procedures and treatments, including biliary drainage and perioperative chemotherapy. Histopathological findings, including tumor size, lymph node involvement, and histological differentiation, were classified according to the 8th edition of the American Joint Committee on Cancer (AJCC) Node Metastasis (TNM) classification system. In this study, four common periampullary malignancies were analyzed, including pancreatic cancer, distal extrahepatic bile duct cancer, ampullary cancer, duodenal and gastric cancer. All cancer identification was based on ICD-9 and ICD-10 coding from CGRD. To evaluate perioperative chemotherapy status, we extracted data on whether patients received chemotherapy within a three-month window before or after the date of surgery. Neoadjuvant chemotherapy was defined as chemotherapy administered within 8 weeks prior to surgery, while adjuvant chemotherapy was defined as chemotherapy initiated within 12 weeks following surgery. This three-month perioperative interval was selected based on clinical relevance and existing guideline recommendations [14,15,16,17].

### 2.2. Nutritional Assessment Using GNRI

All laboratory data for calculating preoperative nutritional status were obtained within 28 days prior to surgery. Patients without a serum albumin measurement or documented body height and/or body weight within this window were excluded. GNRI was calculated using the following formula:

GNRI = 14.89 × [serum albumin] (g/dL) + 41.7 × [present body weight/ideal body weight (kg)](1)

Ideal body weight was determined using the Lorentz equations: 0.75 × height (cm) − 62.5 for males and 0.60 × height (cm) − 40 for females. If a patient’s actual body weight exceeded the ideal body weight, the ratio [current body weight/ideal body weight] was set to 1 [18].

Patients were categorized into three GNRI groups: inferior GNRI status group (GNRI < 82), moderate GNRI status group (82 ≤ GNRI < 98) and superior GNRI status group (GNRI ≥ 98).

While the optimal categorization of the Geriatric Nutritional Risk Index (GNRI) has not been universally standardized, the original GNRI proposed by Bouillanne et al. stratified patients into four groups: major risk (GNRI < 82), moderate risk (GNRI = 82–92), low risk (GNRI = 92–98), and no risk (GNRI ≥ 98) [18]. This four-tier classification remains widely cited and subsequent studies have adapted it by merging moderate risk (GNRI = 82–92) and low risk (GNRI = 92–98) into a single group (GNRI = 82–98), thereby yielding a simplified three-group model (GNRI < 82, GNRI = 82–98 and GNRI ≥ 98). This modification has been adopted in several clinical settings to enhance interpretability while preserving prognostic discrimination [19,20,21]. Accordingly, this three-tier classification was adopted in our study.

### 2.3. Outcome Evaluation

The primary outcomes were overall survival (OS) and recurrence-free survival (RFS) in patients undergoing pancreaticoduodenectomy. OS was defined as the interval from surgery to death. For patients alive at the time of analysis, the OS was censored at the last follow-up. RFS was defined as the time from surgery to tumor recurrence or death from any cause, serving as a key indicator of long-term oncologic outcomes. Secondary surgical outcomes included post-operative complications, length of hospital stay, ICU stay duration, duration of ventilator support, rates of reintubation, ICU readmission, and reoperation, use of total parenteral nutrition (TPN), and mortality at 30 days, 90 days, and one year. Post-operative complications were classified using the Clavien–Dindo (CD) system, with major complications defined as grade III or higher. Documented complications included pancreatic fistula, hemorrhage, delayed gastric emptying requiring TPN, intra-abdominal infection, and cardiopulmonary complications necessitating reintubation or prolonged ventilation.

### 2.4. Statistical Analysis

A propensity score was calculated using logistic regression, including age, sex, cancer type (pancreatic cancer vs. other periampullary malignancies), surgical approach (open vs. minimally invasive), and American Society of Anesthesiologists (ASA) physical status (class I–II vs. III–IV).

Baseline differences between groups were assessed using the chi-square test for categorical variables and the Kruskal–Wallis test for continuous variables owing to the non-normal distribution of data and the presence of three comparison groups. OS was estimated using the Kaplan–Meier method, and survival curves were compared using the log-rank test. Hazard ratios (HRs) with 95% confidence intervals (CIs) were derived from Cox proportional hazards regression models. Both categorical and continuous covariates were incorporated into the models; categorical variables were entered as indicator (dummy) variables, whereas continuous variables were analyzed on a per-unit scale. Univariate analyses were first performed for each variable, and those with a *p*-value < 0.05 were subsequently included in the multivariate Cox regression model. All statistical analyses were conducted using SAS software (v9.4; SAS Institute, Cary, NC, USA). A two-sided *p*-value < 0.05 was considered statistically significant.

## 3. Results

### 3.1. Patient Characteristics

**Figure 1:**
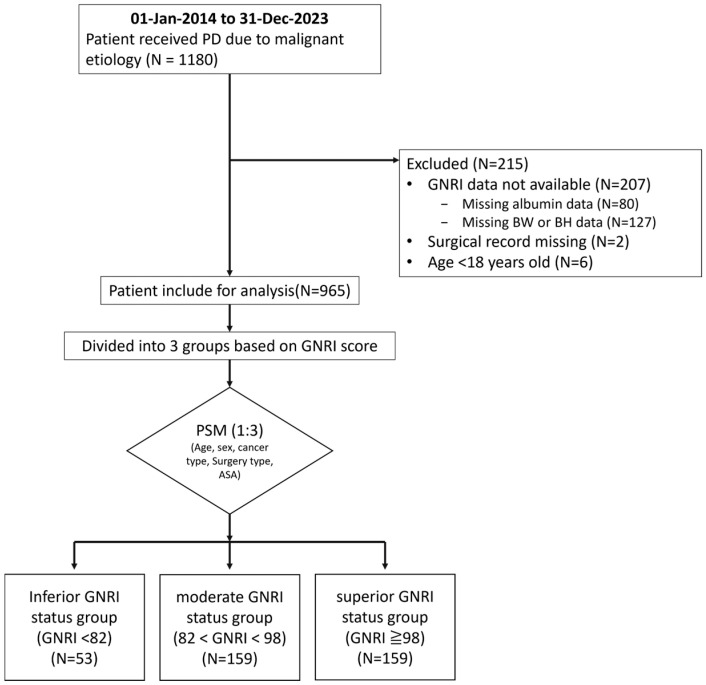
Flow chart of the patient selection process. A total of 1180 patients who underwent pancreaticoduodenectomy were initially identified. After excluding 207 patients with missing GNRI data, along with 2 patients with incomplete surgical records and 6 patients under the age of 18, a total of 965 eligible patients remained. Following 1:3 propensity score matching, 371 patients were included in the final analysis. These were categorized into three groups: 53 in the inferior GNRI status group (GNRI < 82), 159 each in the moderate GNRI status group (82 ≤ GNRI < 98) and the superior GNRI status group (GNRI ≥ 98). GNRI—Geriatric Nutritional Risk Index; BW—body weight; BH—body height; PD—pancreaticoduodenectomy; PSM—propensity score matching.

A total of 1180 patients were included in this study. Of these, 207 were excluded because of missing GNRI data (80 due to missing serum albumin levels and 127 due to missing body weight or body height data), 2 patients were excluded due to incomplete surgical records, and 6 patients younger than 18 years of age were excluded. After 1:3 propensity score matching, 371 patients remained in the final analysis. The inferior GNRI group included 53 patients, whereas the moderate and superior GNRI groups each had 159 patients (Figure 1).

Table 1 and Table A1 (Appendix A) compares clinicopathological characteristics among the three groups before and after matching. After matching, significant differences were observed in body mass index (BMI) (*p* < 0.001), preoperative biliary drainage (*p* = 0.017), preoperative CA19-9 levels (*p* < 0.001), preoperative albumin levels (*p* < 0.001), and preoperative total bilirubin levels (*p* < 0.001). No significant differences were found in cancer type, age, sex, ASA physical status, operative time, or estimated intraoperative blood loss, tumor staging and the percentage of patients receiving perioperative chemotherapy within 3 months. Additionally, hypertension, diabetes mellitus, surgical approach (open vs. minimally invasive), the number of lymph nodes sampled intraoperatively, the number of lymph nodes involved, tumor size, or surgical margin status did not differ significantly among the groups. Notably, only 10 out of 371 patients (2.69%) received neoadjuvant chemotherapy, and 122 out of 371 (32.88%) received adjuvant chemotherapy within 3 months perioperatively. These represent relatively low proportions.

### 3.2. Association Between Different GNRI Groups and Short-Term Outcomes

After propensity score matching, patients with inferior GNRI status had significantly worse outcomes than those with superior GNRI status. They had longer ICU stays (7.22 vs. 4.10 days, *p* < 0.001), prolonged ventilator use (5.02 vs. 2.81 days, *p* = 0.019), higher reintubation rates (15.09% vs. 3.77%, *p* = 0.025), increased TPN use (69.81% vs. 41.51%, *p* = 0.002), and higher reoperation rates (18.87% vs. 4.40%, *p* < 0.001). Mortality was also significantly higher in the inferior GNRI group, including 30-day (11.32% vs. 1.89%, *p* = 0.014), 90-day (24.53% vs. 5.03%, *p* < 0.001) and 1 year mortality (43.40% vs. 14.47%, *p* < 0.001). Post-operative hospital stays, unexpected ICU readmissions, and post-operative complications did not differ significantly among the three groups after matching (Table 2 and Table A2).

### 3.3. Association Between Different GNRI Groups and Survival

#### 3.3.1. Association Between GNRI and Survival

**Figure 2:**
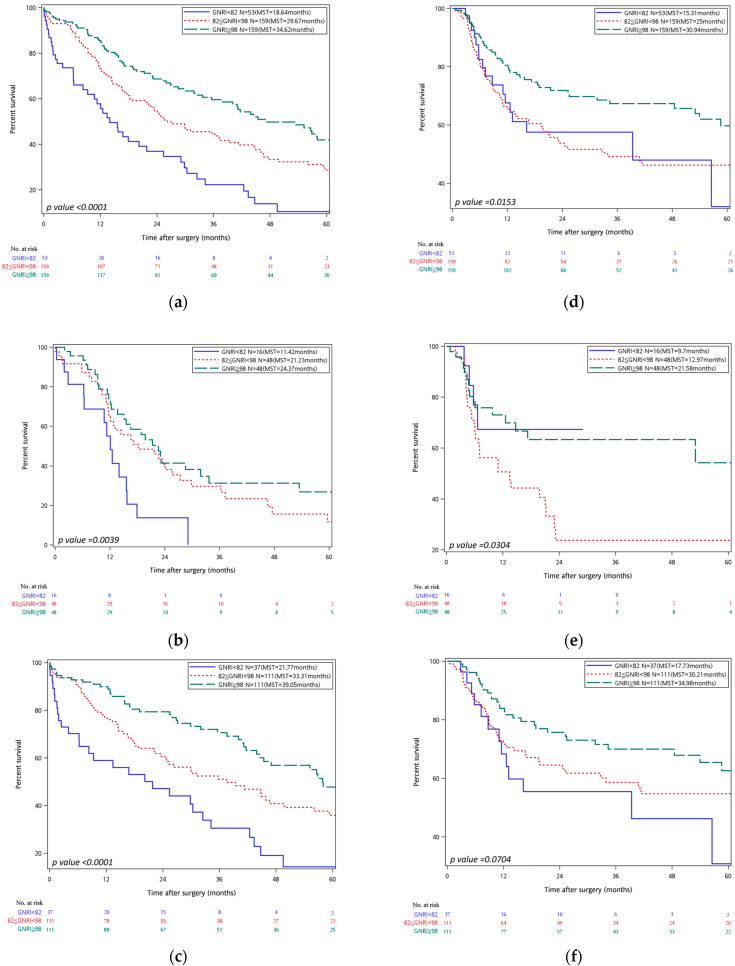
On the left, Kaplan–Meier curves for overall survival stratified by GNRI are shown for all cancer types (**a**) pancreatic cancer, (**b**) and other cancer types (**c**). On the right, Kaplan–Meier curves for recurrence-free survival stratified by GNRI are displayed for all cancer types (**d**), pancreatic cancer (**e**), and other cancer types (**f**). In all panels, GNRI groups are color-coded as follows: inferior GNRI status group (GNRI < 82) (blue), moderate GNRI status group (82 ≤ GNRI < 98) (red-dotted), superior GNRI status group (GNRI ≥ 98) (green-dashed). Median survival times (MSTs) are indicated in each panel. Risk tables are shown beneath each graph.

In the overall population (Figure 2), a higher GNRI score was associated with superior survival outcomes. Patients in the superior GNRI status group (GNRI ≥ 98) achieved a 5-year overall survival (OS) of 18.87% and a median survival time (MST) of 34.62 months, significantly surpassing those in the moderate GNRI status group (82 ≤ GNRI < 98) (5-year OS 14.47%, MST 29.67 months; HR = 1.574, 95% CI 1.153–2.150, *p* = 0.004) and inferior GNRI (GNRI < 82) (5-year OS 3.77%, MST 18.64 months; HR = 2.953, 95% CI 2.009–4.342, *p* < 0.001).

A comparable pattern emerged for recurrence-free survival (RFS): the superior GNRI status group achieved a 5-year RFS of 16.35% and an MST of 30.94 months, outperforming the inferior group (3.77%, 25.0 months; HR = 1.599, 95% CI 1.097–2.331, *p* = 0.015). The difference between superior and moderate GNRI status groups did not reach statistical significance.

#### 3.3.2. Subgroup Analysis: Pancreatic Cancer vs. Other Malignancies

In subgroup analysis of pancreatic cancer patients (N = 112), the superior GNRI cohort demonstrated markedly better OS (5-year OS 10.42%, MST 24.37 months) than the inferior group (0%, 11.42 months; HR = 2.719, 95% CI 1.416–5.220, *p* = 0.003). OS did not differ significantly between superior and moderate GNRI status categories (HR = 1.487, 95% CI 0.900–2.458, *p* = 0.122). For RFS, the superior group again achieved more favorable outcomes (5-year RFS 8.33%, MST 21.58 months) compared with the inferior group (0%, 9.7 months; HR = 1.894, 95% CI 1.019–3.518, *p* = 0.043); no significant difference was observed versus the moderate group.

In patients with malignancies other than pancreatic cancer (N = 259), the superior GNRI status group attained a 5 year OS of 22.52% and an MST of 39.05 months, significantly exceeding both the moderate (18.92%, 33.31 months; HR = 3.164, 95% CI 1.958–5.115, *p* < 0.001) and inferior groups (5.41%, 21.77 months; HR = 1.617, 95% CI 1.087–2.407, *p* = 0.018). RFS did not differ significantly across GNRI groups in this subgroup.

### 3.4. Univariate and Multivariate Cox Regression Analysis

Univariate and multivariate analyses were conducted to identify significant predictors of OS and RFS across subgroups (Table 3 and Table 4). In the overall population, both inferior and moderate GNRI status groups were independently associated with significantly worse OS when compared to the superior GNRI status group. Specifically, patients in the inferior GNRI status group exhibited a HR of 2.869 (95% CI, 1.957–4.205; *p* < 0.001) in univariate analysis and 2.65 (95% CI, 1.706–4.116; *p* < 0.001) in multivariate analysis. Similarly, those in the moderate GNRI status group demonstrated an HR of 1.548 (95% CI, 1.146–2.091; *p* = 0.004) in univariate and 1.768 (95% CI, 1.246–2.511; *p* = 0.001) in multivariate analyses. In addition to GNRI, several perioperative factors were identified as independent risk factors for decreased OS. These included longer operative time (per hour increase: HR = 1.092, 95% CI: 1.024–1.164, *p* = 0.007), higher estimated intraoperative blood loss (per 100 mL increase: HR = 1.041, 95% CI: 1.022–1.059, *p* < 0.001), and larger tumor size (per mm increase: HR = 1.007, 95% CI: 1.001–1.013, *p* = 0.031). Beyond GNRI, lymph node metastasis was strongly associated with increased mortality risk (HR = 2.407, 95% CI: 1.7–3.413, *p* < 0.001). Tumor margin status (HR = 1.613, 95% CI: 1.111–2.340, *p* = 0.012) and receipt of adjuvant chemotherapy within three months post-operatively (HR = 1.346, 95% CI: 1.018–1.778, *p* = 0.037) were significant predictors in the univariate analysis, although neither remained statistically significant in the multivariate model regarding OS. For RFS, only the moderate GNRI status group showed significantly poorer outcomes, with an HR of 1.688 (95% CI, 1.161–2.454; *p* = 0.006) in univariate and 1.865 (95% CI, 1.265–2.751; *p* = 0.002) in multivariate models. Other independent predictors of shorter RFS included an increase in operative time and the presence of lymph node involvement, whereas an increase in age was unexpectedly associated with better RFS. Adjuvant chemotherapy administered within three months post-operatively was significantly associated with worse RFS in the univariate analysis (HR = 2.029, 95% CI: 1.434–2.872, *p* < 0.001), but this association did not remain significant in the multivariate model.

In the pancreatic cancer subgroup, patients with inferior GNRI exhibited significantly worse OS, with an HR of 2.981 (95% CI, 1.531–5.803; *p* = 0.001) in univariate and 2.697 (95% CI, 1.357–5.358; *p* = 0.005) in multivariate analyses. Lymph node involvement and an increase in estimated intraoperative blood loss were also identified as significant predictors of poor OS in this subgroup. For RFS, both lymph node involvement, ASA score greater than three and moderate GNRI status were independent predictors of poorer outcomes. Interestingly, patients in the inferior GNRI status group demonstrated a paradoxically better RFS with no significant difference compared with superior GNRI status group.

Among patients with malignancies other than pancreatic cancer, both inferior and moderate GNRI status categories were significantly associated with worse OS. Inferior GNRI status was linked to an HR of 3.035 (95% CI, 1.888–4.879; *p* < 0.001) in univariate and 2.385 (95% CI, 1.397–4.072; *p* < 0.001) in multivariate analyses, while moderate GNRI status corresponded to an HR of 1.616 (95% CI, 1.106–2.362; *p* = 0.013) in univariate and 1.6 (95% CI, 1.058–2.419; *p* = 0.026) in multivariate models. Additional predictors of poor OS in this group included an ASA score greater than 3, lymph node metastasis, and greater estimated intraoperative blood loss. Lymph node involvement and longer operative time (HR per 1 h increase in operative time = 1.144, 95% CI: 1.05–1.247; *p* = 0.002) are the two independent predictors of poor RFS found in patients with other malignancies.

## 4. Discussion

This study evaluated the relationship between preoperative GNRI scores and post-operative outcomes in patients undergoing pancreaticoduodenectomy (PD).

In this study, a lower GNRI was significantly associated with poorer OS and adverse short-term outcomes. Patients in the inferior GNRI status group had markedly higher 90-day (24.53% versus 5.03%) and 1-year mortality rates (43.4% versus 14.47%), compared to those in the superior GNRI status group (GNRI ≥ 98), with an MST of 18.64 months versus 34.62 months. These findings highlight the impact of malnutrition on prognosis, although part of this association may also reflect the severity of disease- and cancer-related cachexia.

Malnutrition affects up to 70% of patients with upper gastrointestinal (GI) cancers, driven by cancer-related metabolic alterations, pancreatic insufficiency, and impaired gastrointestinal function [1]. Prior studies have demonstrated that sarcopenia is linked to worse OS in pancreatic cancer [22], and lower preoperative GNRI scores have been associated with reduced survival across several cancers, including gastric, colorectal, pancreatic, esophageal, and hepatocellular cancers [23,24,25,26,27,28]. These findings are consistent with our results.

In this study, GNRI was identified as a strong and independent determinant of OS, retaining significance even after adjustment for cancer type, tumor stage, and receipt of adjuvant chemotherapy. Consistent with prior reports, patients with inferior GNRI status experienced higher rates of adverse perioperative outcomes, including prolonged post-operative hospital stay, prolonged ICU stay, unplanned ICU readmission, higher rate of pulmonary complications and greater reliance on parenteral nutrition [13,19]. In our cohort, low GNRI scores were further associated with increased ventilator dependence, higher rates of reintubation, and more frequent reoperation in patient undergoing PD. These short-term complications are known to increase early mortality and exert lasting negative effects on survival [29,30,31,32,33,34], providing a plausible explanation for the inferior long-term outcomes observed in patients in the inferior GNRI status group.

Although adjuvant chemotherapy and preoperative CA19-9 have been reported as prognostic variables for periampullary cancers [34,35,36,37,38,39,40], their effects were not independent in this cohort, whereas lymph node metastasis remained a consistent predictor of OS and RFS [41,42]. The dominant influence of GNRI on survival, therefore, appears to reflect the cumulative burden of short-term complications and high early mortality, which may offset the potential benefit of systemic therapy. Supporting evidence indicates that both low GNRI and incomplete chemotherapy independently predict poor survival [35], and that the adverse impact of major complications on OS is mediated through the omission of adjuvant therapy [34,35,36]. Collectively, these findings suggest that nutritional risk exerts a prognostic effect that may be as important as classical oncologic factors, highlighting GNRI as a simple and objective biomarker that may help identify patients who could benefit from intensified nutritional optimization to improve survival.

Several studies, including randomized controlled trials and meta-analyses, have demonstrated the benefits of nutritional care in patients with moderate-to-severe preoperative malnutrition [43,44,45]. emphasizing the need for preoperative nutritional assessment. Various tools are available to assess nutritional status, and noninvasive biomarkers are gaining popularity owing to their ease of use. Some studies focus exclusively on nutrition, while others also incorporate immune status. However, no consensus has yet been reached regarding a standard preoperative nutritional marker for patients undergoing PD.

Lu et al. compared seven inflammatory–nutritional indicators, including the albumin-to-globulin ratio, Prognostic Nutritional Index (PNI), systemic immune-inflammation index (SII), neutrophil-to-lymphocyte ratio (NLR), platelet-to-lymphocyte ratio (PLR), Nutritional Risk Index (NRI), and GNRI, in pancreatic cancer patients, 51.6% of whom underwent PD. Their findings suggested that PNI was a better predictor of post-operative survival [46]. PNI reflects both nutritional and immune status, as it includes albumin and lymphocyte counts. However, since it incorporates immune parameters, it may not purely reflect nutritional status, which could complicate decisions on nutritional interventions. The NRI requires comparison between a patient’s usual and current weight, which can be difficult to recall, particularly for elderly patients. In contrast, GNRI, based solely on serum albumin and BMI, offers a simpler and more practical method for rapid nutritional assessment.

Sarcopenia is a reliable marker of malnutrition and a known predictor of poor outcomes in patients with cancer [47,48]. However, its assessment using techniques such as bioelectrical impedance analysis (BIA), dual-energy X-ray absorptiometry (DXA), and CT/MRI-based muscle mass or intramuscular adipose tissue content (IMAC) can be expensive and less feasible in routine clinical practice. Other nutritional assessment tools such as the Subjective Global Assessment (SGA), Malnutrition Universal Screening Tool (MUST), Mini Nutritional Assessment (MNA), and Nutritional Risk Screening Tool 2002 (NRS-2002), though effective, require time-consuming interviews, limiting their practicality in high-volume clinical settings.

This study has several limitations. First, as data were obtained from the CGRD, all patient information was de-identified and post-operative complications were identified only through diagnostic codes, limiting access to detailed clinical records. Second, the inferior GNRI status group included relatively few patients, making subgroup analysis underpowered and reducing the statistical robustness. Third, because multiple cancer types were included, staging comparisons were avoided to reduce complexity and maintain a sufficient sample size. However, pancreatic cancer was analyzed separately, as it represented a major subgroup of interest. Fourth, post-operative follow-up of nutritional parameters such as weight loss or changes in nutritional status was not performed, limiting assessment of long-term nutritional impact. Fifth, due to the nature of the CGRD, information on perioperative chemotherapy was limited to whether it was administered within three months pre- or post-operatively, in accordance with guideline recommendations [14]. However, the exact timing of initiation could not be determined. Finally, the study did not compare the GNRI with other nutritional assessment tools, which might have provided additional insights into its predictive value. Future research should incorporate more detailed clinical data, larger cohorts, and longitudinal nutritional follow-up to validate these findings.

## 5. Conclusions

Our study showed that an inferior GNRI status is associated with worse short-term outcomes and reduced overall survival in patients undergoing pancreaticoduodenectomy. These findings highlight the need for preoperative nutritional assessment and suggest that intervention may be warranted, as poor nutritional status is associated with adverse outcomes in this population.

## Figures and Tables

**Table 1:** Baseline characteristics of patients undergoing pancreatoduodenectomy stratified by GNRI categories (n = 371).

	Match (n = 371)			
Characteristics	Inferior GNRI Status GNRI < 82 (n = 53)	Moderate GNRI Status 82 ≤ GNRI < 98 (n = 159)	Superior GNRI Status GNRI ≥ 98 (n = 159)	*p*-Value
Tumor Type				
Pancreatic cancer, n (%)	16 (30.19%)	48 (30.19%)	48 (30.19%)	1
Distal CBD cancer, n (%)	12 (22.64%)	37 (23.27%)	34 (21.38%)	0.921
Ampullary cancer, n (%)	22 (41.51%)	63 (39.62%)	63 (39.62%)	0.967
Duodenal/gastric cancer, n (%)	3 (5.66%)	11 (6.92%)	14 (8.81%)	0.697
Age, year ± SD	69.81 ± 10.26	68.03 ± 10.11	65.82 ± 10.29	0.983
Gender				0.904
Male, n (%)	28 (52.83%)	82 (51.57%)	86 (54.09%)	
Female, n (%)	25 (47.17%)	77 (48.43%)	73 (45.91%)	
BMI, mean ± SD	21.5 ± 2.48	22.33 ± 2.49	23.02 ± 2.63	<0.001 *
Pre-OP biliary drainage, n (%)	28 (52.83%)	90 (56.6%)	65 (40.88%)	0.017 *
Underlying disease				
HTN, n (%)	19 (35.85%)	68 (42.77%)	55 (34.59%)	0.301
DM, n (%)	20 (37.74%)	51 (32.08%)	44 (27.67%)	0.362
Lab data, mean ± SD				
CA19-9 (U/mL), mean ± SD	1284.97 ± 5067.47	660.36 ± 2177.19	492.14 ± 2798.76	<0.001 *
ALB (g/dL), mean ± SD	2.53 ± 0.32	3.37 ± 0.3	4.17 ± 0.28	<0.001 *
T-BIL(mg/dL), mean ± SD	3.44 ± 3.81	3.46 ± 3.92	2.21 ± 3.3	<0.001 *
Surgery Type				
Open, n (%)	52 (98.11%)	155 (97.48%)	155 (97.48%)	0.821
MIS, n (%)	1 (1.89%)	4 (2.52%)	4 (2.52%)	
ASA physical status				0.955
I–II, n (%)	10 (18.87%)	32 (20.13%)	30 (18.87%)	
III–IV, n (%)	43 (81.13%)	127 (79.87%)	129 (81.13%)	
OP time (minutes), mean ± SD	524.95 ± 146.68	515.29 ± 132.66	511.52 ± 155.08	0.516
OP blood loss (mL), mean ± SD	604.34 ± 688.43	492.58 ± 861.86	473.02 ± 733.84	0.058
LN number, mean ± SD	19.36 ± 11.65	20.01 ± 10.81	19.42 ± 10.54	0.747
positive LN number, mean ± SD	1.9 ± 3.66	1.46 ± 2.23	1.33 ± 2.18	0.503
Tumor size (mm), mean ± SD	32.9 ± 23.92	32.97 ± 23.04	28.09 ± 14.66	0.180
Tumor margin				0.713
R0 resection, n (%)	44 (83.02%)	138 (86.79%)	139 (87.42%)	
Not R0 resection, n (%)	9 (16.98%)	21 (13.21%)	20 (12.58%)	
Pathological staging				0.192
Stage I	10 (18.87%)	29 (18.24%)	40 (25.16%)	
Stage II	28 (52.83%)	77 (48.43%)	65 (40.88%)	
Stage III	6 (11.32%)	31 (19.50%)	27 (16.98%)	
Stage IV	4 (7.55%)	3 (1.89%)	6 (3.77%)	
Perioperative chemotherapy in 3 months				
NACT	1 (1.89%)	2 (1.26%)	7 (4.40%)	0.207
ACT	14 (26.42%)	60 (37.74%)	48 (30.19%)	0.2

ALB—albumin; BMI—body mass index; CA19-9—Carbohydrate antigen 19-9; DM—Diabetes mellitus; SD—standard deviation; HTN—hypertension; LN—lymph node; MIS—minimally invasive surgery (laparoscopic or robotic approach); OP—operation or operative; T-BIL—total bilirubin; NACT—neoadjuvant chemotherapy; ACT—adjuvant chemotherapy; * Statistically significant at *p* < 0.05.

**Table 2:** Comparison of short-term outcomes and mortality in patients undergoing pancreatoduodenectomy stratified by GNRI categories (n = 371).

	Match (n = 371)			
Prognostic Outcomes	Inferior GNRI Status GNRI < 82 (n = 53)	Moderate GNRI Status 82 ≤ GNRI < 98 (n = 159)	Superior GNRI Status GNRI ≥ 98 (n = 159)	*p*-Value
Post-operative stays, day(s) ± SD	24.51 ± 11.43	23.57 ± 9.35	27.36 ± 12.7	0.057
Length of ICU stay, day(s) ± SD	7.22 ± 8.79	4.73 ± 5.05	4.1 ± 4.59	0.001 *
Length of ventilator use, day(s) ± SD	5.02 ± 7.44	2.9 ± 4.24	2.81 ± 3.19	0.019 *
Reintubation, n (%)	8 (15.09%)	7 (4.4%)	6 (3.77%)	0.025 *
Readmission to ICU, n (%)	9 (16.98%)	11 (6.92%)	9 (5.66%)	0.337
TPN use, n (%)	37 (69.81%)	74 (46.54%)	66 (41.51%)	0.002 *
≥Grade 3 Complication, n (%)	14 (26.42%)	19 (11.95%)	17 (10.69%)	0.112
Reoperation, n (%)	10 (18.87%)	8 (5.03%)	7 (4.4%)	0.001 *
30-day Mortality, n (%)	6 (11.32%)	7 (4.4%)	3 (1.89%)	0.014 *
90-day Mortality, n (%)	13 (24.53%)	11 (6.92%)	8 (5.03%)	<0.001 *
1-Year Mortality, n (%)	23 (43.4%)	41 (25.79%)	23 (14.47%)	<0.001 *

ICU—intensive care unit; SD—standard deviation; TPN—total parenteral nutrition. * Statistically significant at *p* < 0.05.

**Table 3:** Univariate and multivariate analysis of predictors for overall survival (matched).

Variables		Univariate HR (95% CI)	*p*-Value	Multivariate HR (95% CI)	*p*-Value
ALL					
Age (years) ^§^		1.011 (0.998–1.024)	0.0917		
Sex (female/male)		0.878 (0.671–1.148)	0.343		
BMI (kg/m^2^) ^§^		0.955 (0.905–1.008)	0.098		
ASA physical status (III–IV/I–II)		1.234 (0.87–1.751)	0.239		
Operative time (h) ^§^		1.116 (1.06–1.175)	<0.001 *	1.092 (1.024–1.164)	0.007 *
Lymph node positive (positive/negative)		2.419 (1.835–3.188)	<0.001 *	2.407 (1.699–3.41)	<0.001 *
Operative blood loss (100 mL) ^§^		1.052 (1.037–1.067)	<0.001 *	1.041 (1.022–1.059)	<0.001 *
Tumor size (mm) ^§^		1.007 (1.001–1.012)	0.013 *	1.007 (1.001–1.013)	0.031 *
Tumor margin (not R0 resection/R0 resection)		1.613 (1.111–2.34)	0.012 *	1.133 (0.733–1.744)	0.578
CA19-9 (50 U/mL) ^§^		1.002 (1–1.004)	0.014 *	1.001 (0.999–1.003)	0.365
GNRI	GNRI ≥ 98	reference		reference	
	GNRI = 82–98	1.548 (1.146–2.091)	0.004 *	1.768 (1.246–2.511)	0.001 *
	GNRI < 82	2.869 (1.957–4.205)	<0.001 *	2.65 (1.706–4.116)	<0.001 *
Perioperative chemotherapy in 3 months					
	NACT	0.792 (0.252–2.487)	0.690		
	ACT	1.346 (1.018–1.778)	0.037 *	0.71 (0.499–1.011)	0.058
Pancreatic cancer (N = 112)					
Age (years) ^§^		1.024 (1.002–1.046)	0.034 *	1.024 (1.001–1.048)	0.041 *
Sex (female/male)		1.026 (0.633–1.662)	0.918		
BMI (kg/m^2^) ^§^		0.966 (0.882–1.058)	0.459		
ASA physical status (III–IV/I–II)		0.956 (0.595–1.534)	0.851		
Operative time (h) ^§^		1.081 (0.975–1.199)	0.139		
Lymph node positive (positive/negative)		1.868 (1.154–3.024)	0.011 *	1.715 (1.046–2.814)	0.033 *
Operative blood loss (100 mL) ^§^		1.036 (1.017–1.056)	0.001 *	1.043 (1.022–1.064)	<0.001 *
Tumor size (mm) ^§^		1.004 (0.996–1.012)	0.283		
Tumor margin (not R0 resection/R0 resection)		1.132 (0.68–1.885)	0.633		
CA19-9 (50 U/mL) ^§^		1 (0.997–1.003)	0.908		
GNRI	GNRI ≥ 98	reference		reference	
	GNRI = 82–98	1.373 (0.835–2.259)	0.212	1.401 (0.823–2.382)	0.214
	GNRI < 82	2.981 (1.531–5.803)	0.001 *	2.697 (1.357–5.358)	0.005 *
Perioperative chemotherapy in 3 months					
	NACT	0.493 (0.12–2.017)	0.325		
	ACT	0.934 (0.595–1.466)	0.765		
Other Malignancy(N = 259)					
Age (years) ^§^		1.022 (1.004–1.04)	0.018 *	1.009 (0.988–1.03)	0.404
Sex (female/male)		0.982 (0.702–1.372)	0.914		
BMI (kg/m^2^) ^§^		0.957 (0.896–1.022)	0.187		
ASA physical status (III–IV/I–II)		2.283 (1.277–4.082)	0.005 *	2.089 (1.064–4.104)	0.033 *
Operative time (h) ^§^		1.101 (1.033–1.173)	0.003 *	1.082 (0.999–1.173)	0.054
Lymph node positive (positive/negative)		2.44 (1.734–3.434)	<0.001 *	2.417 (1.667–3.503)	<0.001 *
Operative blood loss (100 mL) ^§^		1.083 (1.046–1.121)	<0.001 *	1.061 (1.019–1.106)	0.004 *
Tumor size (mm) ^§^		1.005 (0.997–1.013)	0.197	1 (1–1)	0.142
Tumor margin (not R0 resection/R0 resection)		1.635 (0.921–2.902)	0.093		
CA19-9 (50 U/mL) ^§^		1.003 (1.001–1.006)	0.006 *	1.002 (0.999–1.005)	0.142
GNRI	GNRI ≥ 98	reference		reference	
	GNRI = 82–98	1.616 (1.106–2.362)	0.013 *	1.6 (1.058–2.419)	0.026 *
	GNRI < 82	3.035 (1.888–4.879)	<0.001 *	2.385 (1.397–4.072)	<0.001 *
Perioperative chemotherapy in 3 months					
	NACT	0.976 (0.136–7.017)	0.980		
	ACT	1.273 (0.872–1.859)	0.211		

* Statistically significant at *p* < 0.05; ^§^ Kruskal–Wallis test was used for continuous variables; NACT, neoadjuvant chemotherapy; ACT, adjuvant chemotherapy.

**Table 4:** Univariate and multivariate analysis of predictors for recurrence-free survival (matched).

Variables		Univariate HR (95% CI)	*p*-Value	Multivariate HR (95% CI)	*p*-Value
ALL					
Age (years) ^§^		0.98 (0.966–0.995)	0.001 *	0.978 (0.963–0.994)	0.006 *
Sex (female/male)		0.934 (0.662–1.318)	0.699		
BMI (kg/m^2^) ^§^		0.949 (0.883–1.02)	0.157		
ASA physical status (III–IV/I–II)		0.688 (0.463–1.021)	0.063		
Operative time (h) ^§^		1.137 (1.065–1.213)	<0.001 *	1.159 (1.079–1.245)	<0.001 *
Lymph node positive (positive/negative)		4.434 (3.028–6.493)	<0.001 *	4.583 (3.019–6.956)	<0.001 *
Operative blood loss (100 mL) ^§^		1.023 (0.995–1.052)	0.106		
Tumor size (mm) ^§^		1.005 (0.998–1.013)	0.168		
Tumor margin (not R0 resection/R0 resection)		0.88 (0.496–1.562)	0.662		
CA19-9 (50 U/mL) ^§^		1.001 (0.999–1.004)	0.168		
GNRI	GNRI ≥ 98	reference		reference	
	GNRI = 82–98	1.688 (1.161–2.454)	0.006 *	1.865 (1.265–2.751)	0.002 *
	GNRI < 82	1.679 (0.962–2.929)	0.068	1.681 (0.955–2.957)	0.072
Perioperative chemotherapy in 3 months					
	NACT	0.61 (0.151–2.471)	0.489		
	ACT	2.029 (1.434–2.872)	<0.001 *	0.846 (0.573–1.249)	0.4
Pancreatic cancer (N = 112)					
Age (years) ^§^		0.981 (0.957–1.006)	0.139		
Sex (female/male)		1.53 (0.847–2.764)	0.159		
BMI (kg/m^2^) ^§^		0.981 (0.827–1.047)	0.232		
ASA physical status (III–IV/I–II)		0.445 (0.25–0.793)	0.006 *	0.438 (0.244–0.786)	0.006 *
Operative time (h) ^§^		1.136 (0.998–1.293)	0.054		
Lymph node positive (positive/negative)		4.87 (2.26–10.492)	<0.001 *	4.651 (2.146–10.081)	<0.001 *
Operative blood loss (100 mL) ^§^		1 (0.963–1.039)	0.986		
Tumor size (mm) ^§^		0.997 (0.981–1.012)	0.682		
Tumor margin (not R0 resection/R0 resection)		0.625 (0292–1.339)	0.227		
CA19-9 (50 U/mL) ^§^		0.999 (0.995–1.004)	0.805		
GNRI	GNRI ≥ 98	reference		reference	
	GNRI = 82–98	2.163 (1.162–4.027)	0.015 *	1.922 (1.03–3.586)	0.04 *
	GNRI < 82	1.044 (0.347–3.144)	0.939	0.736 (0.244–2.217)	0.586
Perioperative chemotherapy in 3 months					
	NACT	0.268 (0.037–1.947)	0.193		
	ACT	1.779 (0.963–3.29)	0.066		
Other Malignancy(N = 259)					
Age (years) ^§^		0.988 (0.967–1.008)	0.231		
Sex (female/male)		0.904 (0.587–1.391)	0.646		
BMI (kg/m^2^) ^§^		0.961 (0.88–1.051)	0.387		
ASA physical status (III–IV/I–II)		1.31 (0.693–2.473)	0.406		
Operative time (h) ^§^		1.113 (1.026–1.208)	0.01 *	1.144 (1.05–1.247)	0.002 *
Lymph node positive (positive/negative)		3.907 (2.49–6.129)	<0.001 *	4.035 (2.413–6.747)	<0.001 *
Operative blood loss (100 mL)		1.043 (0.989–1.1)	0.119		
Tumor size (mm) ^§^		1.007 (0.998–1.017)	0.138		
Tumor margin (not R0 resection/R0 resection)		0.88 (0.356–2.177)	0.783		
CA19-9 (50 U/mL) ^§^		1.003 (1–1.006)	0.027 *	1.001 (0.998–1.004)	0.385
GNRI	GNRI ≥ 98	reference		reference	
	GNRI = 82–98	1.471 (0.918–2.357)	0.109	1.438 (0.88–2.349)	0.147
	GNRI < 82	2.022 (1.056–3.87)	0.034 *	1.692 (0.861–3.326)	0.127
Perioperative chemotherapy in 3 months					
	NACT	1.143 (0.159–8.239)	0.894		
	ACT	1.788 (1.132–2.823)	0.013 *	0.891 (0.527–1.504)	0.665

* Statistically significant at *p* < 0.05; ^§^ Kruskal–Wallis test was used for continuous variables; NACT, neoadjuvant chemotherapy; ACT, adjuvant chemotherapy.

## Data Availability

The datasets generated and analyzed in this study are derived from the Chang Gung Research Database (CGRD) and are de-identified. Due to institutional policy and data use agreements, the raw data is not publicly available.

## Abbreviations

The following abbreviations are used in this manuscript:

PD	Pancreaticoduodenectomy
GNRI	Geriatric Nutritional Risk Index
OS	Overall Survival
RFS	Recurrence-Free Survival
HR	Hazard ratio
CI	Confidence Interval
MST	Median Survival Time
ACT	Adjuvant chemotherapy
NACT	Neoadjuvant chemotherapy
LN	Lymph node
ICU	Intensive Care Unit
TPN	Total Parenteral Nutrition
ASA	American Society of Anesthesiologists
AJCC	American Joint Committee on Cancer
PSM	Propensity Score Matching
CGRD	Chang Gung Research Database

## Appendix A

**Table A1:** Baseline characteristics of patients undergoing pancreaticoduodenectomy according to GNRI categories before and after propensity score matching (PSM).

	Cohort (N = 1203)				Match (N = 371)			
Characteristics	Inferior GNRI < 82 (n = 67)	Moderate 82 ≤ GNRI < 98 (n = 544)	Superior GNRI ≥ 98 (n = 592)	*p*-Value	Inferior GNRI < 82 (n = 53)	Moderate 82 ≤ GNRI < 98 (n = 159)	Superior GNRI ≥ 98 (n = 159)	*p*-Value
Tumor Type								
Pancreatic cancer, n (%)	16 (23.88%)	299 (42.1%)	242 (40.88%)	0.0158	16 (30.19%)	48 (30.19%)	48 (30.19%)	1
Distal CBD cancer, n (%)	12 (17.91%)	76 (13.97%)	61 (10.3%)	0.0637	12 (22.64%)	37 (23.27%)	34 (21.38%)	0.9206
Ampullary cancer, n (%)	22 (32.84%)	140 (25.74%)	106 (17.91%)	0.0007	22 (41.51%)	63 (39.62%)	63 (39.62%)	0.9668
Duodenal/gastric cancer, n (%)	3 (4.48%)	25 (4.6%)	33 (5.57%)	0.7349	3 (5.66%)	11 (6.92%)	14 (8.81%)	0.6973
Benign, n (%)	14 (20.9%)	74 (13.6%)	150 (25.34%)	<0.0001				
Age, year ± SD	67.06 ± 13.62	66.08 ± 10.58	60.89 ± 11.69	<0.0001	69.81 ± 10.26	68.03 ± 10.11	65.82 ± 10.29	0.9834
Gender				0.1015				0.904
Male, n (%)	39 (58.21%)	330 (60.66%)	322 (54.39%)		28 (52.83%)	82 (51.57%)	86 (54.09%)	
Female, n (%)	28 (41.79%)	214 (39.34%)	270 (45.61%)		25 (47.17%)	77 (48.43%)	73 (45.91%)	
BMI, mean ± SD	21.37 ± 2.29	22.21 ± 2.47	22.85 ± 2.72	<0.0001	21.5 ± 2.48	22.33 ± 2.49	23.02 ± 2.63	0.0004
Pre-OP biliary drainage, n (%)	31 (46.27%)	274 (50.37%)	176 (29.73)	<0.0001	28 (52.83%)	90 (56.6%)	65 (40.88%)	0.0169
Underlying disease								
HTN, n (%)	23 (34.33%)	187 (34.38%)	175 (29.56%)	0.2023	19 (35.85%)	68 (42.77%)	55 (34.59%)	0.3007
DM, n (%)	22 (32.84%)	152 (27.94%)	163 (27.53%)	0.6565	20 (37.74%)	51 (32.08%)	44 (27.67%)	0.3618
Lab data, mean ± SD								
CA19-9 (ng/mL), mean ± SD	1201.93 ± 4828.04	648.88 ± 2571.32	306.85 ± 1563.45	<0.0001	1284.97 ± 5067.47	660.36 ± 2177.19	492.14 ± 2798.76	<0.0001
ALB (g/dL), mean ± SD	2.5 ± 0.35	3.4 ± 0.3	4.24 ± 0.3	<0.0001	2.53 ± 0.32	3.37 ± 0.3	4.17 ± 0.28	<0.0001
T-BIL (mg/dL), mean ± SD	3.36 ± 3.64	3.4 ± 3.91	1.81 ±2.66	<0.0001	3.44 ± 3.81	3.46 ± 3.92	2.21 ± 3.3	<0.0001
Surgery Type				0.1979				
Open, n (%)		525 (96.51%)	599 (94.43%)		52 (98.11%)	155 (97.48%)	155 (97.48%)	0.8207
MIS, n (%)		19 (3.49%)	33 (5.57%)		1 (1.89%)	4 (2.52%)	4 (2.52%)	
ASA physical status				0.0017				0.9551
I–II, n (%)	12 (17.91%)	106 (19.49%)	166 (28.04%)		10 (18.87%)	32 (20.13%)	30 (18.87%)	
III–IV, n (%)	55 (82.09%)	438 (80.51%)	426 (71.96%)		43 (81.13%)	127 (79.87%)	129 (81.13%)	
OP time (minutes), mean ± SD	520.64 ± 147.07	525.75 ± 134.98	517.96 ± 145.16	0.2332	524.95 ± 146.68	515.29 ± 132.66	511.52 ± 155.08	0.5163
OP blood loss (mL), mean ± SD	791.42 ± 982.41	549.52 ± 836.49	420.24 ± 581.51	<0.0001	604.34 ± 688.43	492.58 ± 861.86	473.02 ± 733.84	0.0577
LN number, mean ± SD	19.35 ± 11.48	19.87 ± 10.22	19.19 ± 10.39	0.5356	19.36 ± 11.65	20.01 ± 10.81	19.42 ± 10.54	0.7468
positive LN number, mean ± SD	1.96 ± 3.60	1.90 ± 2.49	1.85 ± 2.95	0.3376	1.9 ± 3.66	1.46 ± 2.23	1.33 ± 2.18	0.5032
Tumor size (mm), mean ± SD	33.10 ± 23.57	31.97 ± 18.26	30.04 ± 16.27	0.256	32.9 ± 23.92	32.97 ± 23.04	28.09 ± 14.66	0.1799
Tumor margin				0.1131				0.7125
R0 resection, n (%)	58 (86.57%)	444 (81.62%)	509 (85.98%)		44 (83.02%)	138 (86.79%)	139 (87.42%)	
Not R0 resection, n (%)	9 (13.43%)	100 (18.38%)	83 (14.02%)		9 (16.98%)	21 (13.21%)	20 (12.58%)	
Pathological staging				0.014				0.192
Stage I	10 (14.93%)	87 (15.99%)	108 (18.24%)		10 (18.87%)	29 (18.24%)	40 (25.16%)	
Stage II	29 (43.28%)	240 (44.12%)	193 (32.60%)		28 (52.83%)	77 (48.43%)	65 (40.88%)	
Stage III	7 (10.45%)	109 (20.04%)	79 (13.34%)		6 (11.32%)	31 (19.50%)	27 (16.98%)	
Stage IV	4 (5.97%)	11 (2.02%)	19 (3.21%)		4 (7.55%)	3 (1.89%)	6 (3.77%)	
Perioperative chemotherapy in 3 months								
NACT	2 (2.99%)	11 (2.02%)	30 (5.07%)	0.021	1 (1.89%)	2 (1.26%)	7 (4.40%)	0.207
ACT	16 (23.88%)	212 (38.97%)	214 (36.15)	0.049	14 (26.42%)	60 (37.74%)	48 (30.19%)	0.2

BMI, body mass index; SD, standard deviation; OP, operation or operative; HTN, hypertension; DM, Diabetes mellitus; CA19-9, Carbohydrate antigen 19-9; ALB, albumin; T-BIL, total bilirubin; MIS, minimally invasive surgery (laparoscopic or robotic approach); LN, lymph node; NACT, neoadjuvant chemotherapy; ACT, adjuvant chemotherapy.

**Table A2:** Comparison of short-term outcomes and mortality in patients undergoing pancreatoduodenec-tomy stratified by GNRI categories before and after propensity score matching (PSM).

	Cohort (n = 1203)				Match (n = 371)			
Prognostic Outcomes	GNRI < 82 (n = 67)	82 ≤ GNRI < 98 (n = 544)	GNRI ≥ 98 (n = 592)	*p*-Value	GNRI < 82 (n = 53)	82 ≤ GNRI < 98 (n = 159)	GNRI ≥ 98 (n = 159)	*p*-Value
Post-operative stays, day(s) ± SD	24.28 ± 12.6	23.78 ± 10.4	24.14 ± 11.9	0.682	24.51 ± 11.43	23.57 ± 9.35	27.36 ± 12.7	0.0573
Length of ICU stay, day(s) ± SD	7.55 ± 8.53	4.74 ± 6.26	3.54 ± 3.66	<0.0001	7.22 ± 8.79	4.73 ± 5.05	4.1 ± 4.59	0.0007
Length of ventilator use, day(s) ± SD	5.4 ± 7.32	3.18 ± 5.41	2.49 ± 2.61	<0.0001	5.02 ± 7.44	2.9 ± 4.24	2.81 ± 3.19	0.0191
Reintubation, n (%)	9 (13.43%)	18 (3.31%)	17 (2.87%)	<0.0001	8 (15.09%)	7 (4.4%)	6 (3.77%)	0.025
Readmission to ICU, n (%)	10 (13.43%)	30 (5.51%)	25 (4.22%)	0.0012	9 (16.98%)	11 (6.92%)	9 (5.66%)	0.3365
TPN use, n (%)	46 (68.66%)	229 (42.1%)	219 (36.99%)	<0.0001	37 (69.81%)	74 (46.54%)	66 (41.51%)	0.0016
≥Grade 3 Complication, n (%)	20 (29.85%)	55 (10.11%)	47(7.94%)	<0.0001	14 (26.42%)	19 (11.95%)	17 (10.69%)	0.112
Reoperation, n (%)	14 (20.9%)	30 (5.51%)	25 (4.22%)	<0.0001	10 (18.87%)	8 (5.03%)	7 (4.4%)	0.0007
30-day Mortality, n (%)	10 (14.93%)	12 (2.21%)	9 (1.52%)	<0.0001	6 (11.32%)	7 (4.4%)	3 (1.89%)	0.0137
90-day Mortality, n (%)	18 (26.87%)	30 (5.51%)	18 (3.04%)	<0.0001	13 (24.53%)	11 (6.92%)	8 (5.03%)	<0.0001
1-Year Mortality, n (%)	28 (41.79%)	138 (25.37%)	84 (14.19%)	<0.0001	23 (43.4%)	41 (25.79%)	23 (14.47%)	<0.0001

SD, standard deviation; ICU, intensive care unit; TPN, total parenteral nutrition.

**Table A3:** Adjuvant chemotherapy (≤12 weeks) across GNRI groups among stage II–IV patients. To further investigate whether GNRI status influenced the likelihood of receiving adjuvant chemotherapy, we conducted a subgroup analysis restricted to patients with stage II or higher disease from each GNRI status group. A chi-square test revealed no statistically significant difference in adjuvant chemotherapy receipt among these groups.

GNRI Status	Stage II or Higher (N)	ACT Within 3 Months, n (%)	No ACT Within 3 Months, n (%)
Inferior GNRI status (GNRI < 82)	38	14 (36.8%) *	24 (63.2%) *
moderate GNRI status (82 ≤ GNRI < 98)	111	60 (54.1%) *	51 (45.9%) *
superior GNRI status (GNRI ≥ 98)	98	48 (49%) *	50 (51.0%) *
total	247	122 (49.4%) *	125 (50.6%) *

* Denominators are restricted to patients with pathologic stage II–IV (AJCC 8th). Receipt of adjuvant chemotherapy was derived from CGRD treatment records and may include a small number of stage I cases; thus “ACT received” counts reflect all recorded recipients irrespective of final pathologic stage. ACT, adjuvant chemotherapy. *Statistical tests:* Pearson’s χ^2^(2) = 3.3662, *p*-value = 0.185799. The result is not significant at *p* < 0.05.
